# Surgery of Gall-Bladder and Bile-Ducts

**Published:** 1903-03-14

**Authors:** 


### SURGERY OF GALL-BLADDER AND BILE-DUCTS.

In a paper read before the Academy of Medicine in New York, in December of last year, Mr. Mayo[1] dealt with the present status of the surgery of the gall-bladder and bile-ducts. The medical profession, he considers, has been somewhat slow to accept the modern view of gall-stone disease, and, although introduced at about the same time as the "appendicitis problem," as he calls it, it is far from as definite a solution. The reasons for this are that in gall-stone disease the patient is usually advanced in years, and often, by reason of degenerative lesions or excess of adipose tissue, not a good subject for operation; and, again, death does not often come with that tragic suddenness which ofttimes characterises appendicitis. But all of these considerations are of minor importance when compared to the major obstacle to a proper understanding of this common malady, which is by no means the innocent affection our predecessors taught us to believe. It is the "slumbering gall-stone" which stands in the way. He quotes statistics from several authors illustrating the frequeney with which gall-stones are discovered post-mortem. Thus Bevan found that 16 per cent. of the cadavers at the Rush dissecting rooms had gall-stones. It should be remembered that these statistics are open to the error introduced by the fact that they are mainly or entirely drawn from patients in hospitals and similar institutions. Dr. Mayo thinks that these are the very subjects in whom the two cardinal factors of gall-stone formation are most likely to be present, namely, defective circulation of bile, and that mild infection aptly termed "stone-building catarrh." If only 5 per cent. of adults have gall-stones and 95 per cent. of these cases have no symptoms, it makes the physician pause before recommending an operation. The chance of picking out the surgical case would be only one in twenty. Such an objection is only theoretical because the physician would only have to deal with cases in which symptoms were present. But the proportion of actually "slumbering" gall-stones is much less than is commonly thought; the term is a relative one and means that the disturbance is so slight that the cause is unsuspected. Medical men have grown so accustomed to looking upon the colic as the one symptom of gall-stones that they often, if not usually, overlook its more chronic manifestations. What causes latent calculi to become active? There are two factors—(1) infection of gall-bladder, (2) interference with drainage from it. The lack of free drainage may be due either to a calculus blocking the cystic duct or to inflammatory swelling of the ducts due to infection. The resulting disturbance depends upon the acuteness of the infection, and upon the ability of the organ to distend. The gall-bladder has the advantage over the appendix in this respect, as well as a far better blood supply. Rupture and gangrene are therefore rare, and sudden death from fatal perforation of the gall-bladder does not often occur. Normal bile is free from bacteria, but the calculous gall-bladder was found by Mikulicz to contain bacteria in 18 out of 23 cases. Under favourable circumstances disturbance due to gall-stones may subside, and it is this phenomenon that causes the divergence of opinion between the advocates of early surgical interference and those who rely on medical treatment so long as that is sufficient to hold the symptoms in abeyance. It is the case of chronic and relapsing appendicitis over again, save that in gall-stone disease the patient is as a rule older than, and not such a fit subject for operation as the sufferer from appendicitis. There seems to be a danger of operative intemperance on the one hand, and on the other an equally dangerous tendency to procrastination until the disease has either advanced too far to benefit from operation, or so far as to render the perils of active interference far greater than they would have been at an earlier stage of the disease. The results of early operation for gall-stones is remarkably good. Dr. Mayo and his brother have had between them about 250 cases of this kind with a mortality of less than 1 per cent. In over 2,000 of such operations in the hands of six surgeons, there was not a single instance of re-formation of the gall-stones. In this field of surgery delay breeds misfortune. Complications are due to changes in the wall of the gall-bladder or involvement of the bile-ducts, and the calculi may then become but an incident in the pathological process which they initiated. A thickened, shrunken chronically-inflamed gall-bladder, or one adherent to surrounding viscera, especially to the duodenum, may be a prolonged source of distress and danger to the patient, while the difficulties and dangers of operation in such a condition are very greatly enhanced. Moreover, it is in this class of case that cancer of the gall-bladder is most likely to supervene. In 454 operated cases Dr. Mayo found cancer of the gall-bladder or bile-ducts in 21 cases (5 per cent.), and in nearly all a distinct history of previous colics was elicited. In every case of biliary cancer in which the gall-bladder was examined it was found to contain stones. The chronic irritation of calculi in a gall-bladder in which inflammatory changes have taken place seems to be the usual precancerous condition. Dr. Mayo has never operated on a case in which the patient brought him stones detected in the fæces without finding more stones. Occasionally cases are met with where a stone has remained for years in the common or hepatic duct and has given rise to comparatively little trouble. Infection of the liver ducts introduces an element of uncertainty in the prognosis and is the commonest cause of fatality after operation. In these cases cessation of liver function from degeneration of the liver parenchyma may be present. Quincke has found that infection by the colon bacillus is more liable to cause acute liver degeneration than infection by the ordinary pyogenic organisms. Every gall-bladder containing calculi may be looked upon as infected, and therefore immediate suture of the organ and of the abdominal wound is dangerous.

[1] New York Med. Rec., Feb. 21.

